# Enhanced Flexibility and Reusability through State Machine-Based Architectures for Multisensor Intelligent Robotics

**DOI:** 10.3390/s17061249

**Published:** 2017-05-31

**Authors:** Héctor Herrero, Jose Luis Outón, Mildred Puerto, Damien Sallé, Karmele López de Ipiña

**Affiliations:** 1 Tecnalia Research and Innovation, Industry and Transport Division, San Sebastián 20009, Spain; joseluis.outon@tecnalia.com (J.L.O.); mildred.puerto@tecnalia.com (M.P.); damien.salle@tecnalia.com (D.S.); 2 Department of Systems Engineering and Automation, Universidad del País Vasco/Euskal Herriko Unibertsitatea, EleKin Research Group, San Sebastián 20009, Spain; karmele.ipina@ehu.eus

**Keywords:** intelligent robotics, flexibility, reusability, multisensor, state machine, software architecture, computer vision

## Abstract

This paper presents a state machine-based architecture, which enhances the flexibility and reusability of industrial robots, more concretely dual-arm multisensor robots. The proposed architecture, in addition to allowing absolute control of the execution, eases the programming of new applications by increasing the reusability of the developed modules. Through an easy-to-use graphical user interface, operators are able to create, modify, reuse and maintain industrial processes, increasing the flexibility of the cell. Moreover, the proposed approach is applied in a real use case in order to demonstrate its capabilities and feasibility in industrial environments. A comparative analysis is presented for evaluating the presented approach versus traditional robot programming techniques.

## 1. Introduction

An analysis [1] of the current situation in manufacturing plants highlights three major trends:

- An ever-increasing customization of products and short lifecycle, which require an increase in the flexibility of the production means (one unique system must handle all of the product diversity and operations) [2,3]. Robots fit perfect into this topic due to their versatility; robot programs can adapt to the customizations of the products.
- A large variation in production volumes, which requires an increase in the reconfigurability of production (one system for one product/task within recombinable production lines) [2,4]. Robotic mobile platforms play an important role in this trend; easy to move robots are necessary in some production chains where production volumes change frequently.
- Limited access to skilled operators due to an aging workforce, changes in education and an ever-faster technology development. This requires new solutions to assist operators and provide collaborative work environments [5]. Collaborative robotics are being developed for this topic.

The research addressed in this paper focuses on the first trend: the need for highly flexible and intelligent robotic systems. Despite the large effort in the research community, large companies, as well as small and medium enterprises (SME) still do not have appropriate software tools and solutions to react rapidly with economic viability for an interesting return of investment for the automation of their processes. The direct consequence is that production operations are mostly performed manually, with high operation costs that endanger those companies with respect to lower wage countries. This research is thus oriented toward developing and providing a software ecosystem that allows for a rapid and efficient programming of production processes, providing the required flexibility and permitting an effective integration of auxiliary sensors and artificial vision systems. Even if this approach is generic and applicable to industrial manipulators, this paper will be focused on dual-arm multisensor robotic operations.

The dual-arm robots provide more dexterity, in addition to the advantage that they can be used in the existing workstations. Due to these arguments, the dual-arm robot deployment is growing year by year, not only in large multinationals, but also in SMEs. Sector experts [2,6] affirm investments for robot deployment are amortized in 1–2 years; however, this information cannot be extrapolated to all cases. However, applications with short production batches, environments prone to many changes and processes that need human-robot collaboration or special environment supervision do not comply with this trend. Dual-arm robots are being introduced in such contexts. The growth of dual-arm systems [7] is resulting in many efforts made by robotic researchers to manage them. Programming, coordinating and supervising bi-manual robots is a need that is increasingly being demanded by the community; even more with the rise of collaborative robots, which have to integrate different sensors for cell supervising and monitoring [8,9]. In this scenario, the need for actuation when external signals are received becomes essential, e.g., a person enters the workspace of the robot, and the robot must stop its movement and adapt its behavior.

In this paper, we present an approach to alleviate the challenges that can be identified for dual-arm robotic programming. The presented framework eases the deployment of industrial applications and allows managing the execution control, increasing the reliability and traceability of the system (Section 2). To ease the deployment of this kind of application, we present how the framework can integrate skill-based programming. For understanding the advantages, the assembly operation of an aeronautical part is detailed. Moreover, an evaluation of the architecture is presented (Section 3). Finally, we present the discussion, conclusions and future work (Section 4 and Section 5).

## 2. Materials and Methods

### 2.1. State Machine-Based Execution Coordination for Dual-Arm Robots

Traditional robot programming is still not very flexible; thus, the dual-arm programming suffers the same problems. In the industry, smaller and smaller batches are ordered, and as a consequence, the costs of reprogramming the robots grow. Even though there are usually different parts, the process is very similar, e.g., assembling parts with different types of screws. In this case, the assembly operation is the same; only the screw size, type or position is changing. Those tasks can be modeled; the key is to be able to subdivide a task (screw operation) into smaller operations (robot movement, end-effector actuation, etc.). Then, re-using these tasks can be made parametrizing correctly the corresponding suboperations without needing to reprogram the whole task. Grouping the robot basic movements (primitives) according to tasks or skills is an alternative that many authors have followed [10,11,12,13,14].

One of the most relevant issues in dual-arm robotic programming, especially for industrial applications, is the lack of powerful and easy to use graphical user interfaces [15]. An easy to configure graphical user interface (GUI), which allows the previously-mentioned skill-based programming, will enable operators to program and maintain the industrial processes. This, in addition to the workers feeling a part of the automation process, will also contribute to reduce the costs of the robotic systems’ deployment.

Regarding the execution control, state machines can address dual-arm challenges. These tools are commonly used for general-purpose processes, and in particular, they have been extensively adopted by the robotics community. In this aspect, the work made by different authors combining finite state machines with knowledge and skills is very relevant [16,17,18]. State machines are an easy way for describing behaviors and for modeling how components react to different external and internal stimuli [19,20]. In this area, there are different implementation alternatives, e.g., there are many projects using Orocos rFSM [21]. rFSM is a small and powerful state-chart implementation designed for coordinating complex systems, such as robots. SMACH [22] is another implementation of state machines. It can be defined as a task-level architecture for rapidly creating complex robot behavior. In this work, SMACH has been selected for implementing the state machine. One of the reasons is because SMACH can be used under the ROS (Robot Operating System) [23,24], which is a flexible framework for writing robot software. ROS is a collection of tools, libraries and conventions [25] that aims to simplify the task of creating complex and robust robot behavior across a wide variety of robotic platforms [26]. As a complementary element to the execution control, multi-agent systems can be useful for decision making in coordination and synchronization tasks [27,28].

#### 2.1.1. Proposed Architecture

**Figure 1:**
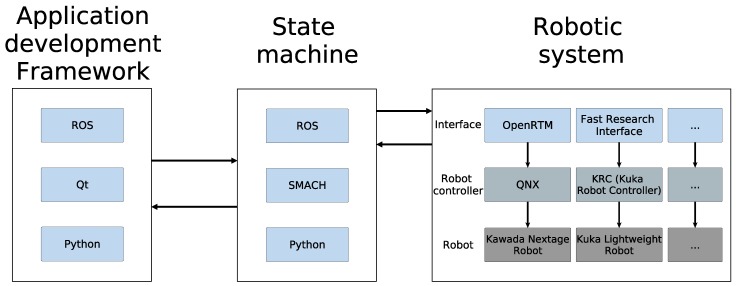
Proposed overall architecture. The figure shows how it is divided into three levels.

As illustrated in Figure 1, the proposed state machine interconnects the application development framework (graphical user interface) and the robotic lower level control system. The presented state machine is composed of different states where each state corresponds to one of the basic operations that the robot can execute. Basic operations are considered the functions or commands that by themselves are able to achieve a goal, e.g., Cartesian point to point interpolation. It can be understood as a robot API (application programming interface). Following the program provided by the user, the active state triggers its corresponding state to execute the necessary functions.

**Figure 2:**
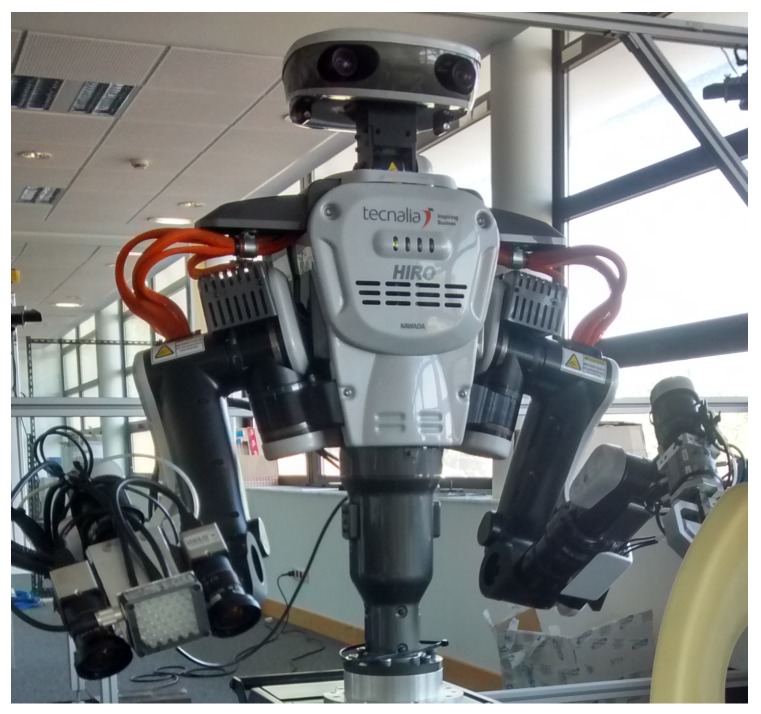
Nextage Open Robot where all developments are being tested.

In this research, all of the prototypes are being tested and validated in a dual-arm robot, specifically in a Kawada Nextage Open Robot (Figure 2). This robot has humanoid aspects, with two arms of 6 degrees of freedom (DOF) attached to a rotatory torso; it is equipped with a 2-DOF head, which incorporates the stereo vision system. In conclusion, it is a 15-DOF robot managed by a single controller. In order to obtain more precision, other stereo vision systems have been added to each wrist.

As is detailed in Section 3.1, the applications are composed of tasks, and these in turn are composed of primitives (or previously-mentioned basic operations), which are translated to states. On the one hand, the execution engine triggers state changes at the low level. On the other hand, in the case of the Kawada Nextage Open robot, the states are connected to the robotic system through an OpenRTM bridge [29]. Even so, it should not be forgotten that ROS allows hardware independence, and changing the bridge properly, another robotic system can be used (for example, Orocos or the Fast Research Interface [30] to interface a Kuka LWR with the proposed architecture).

This combination of the application development framework and a low level state machine allows us to considerably improve the flexibility and hardiness, make the programming easier, achieve hardware independence and environment control, resulting in a more industry-oriented solution.

#### 2.1.2. Core Description

**Figure 3:**
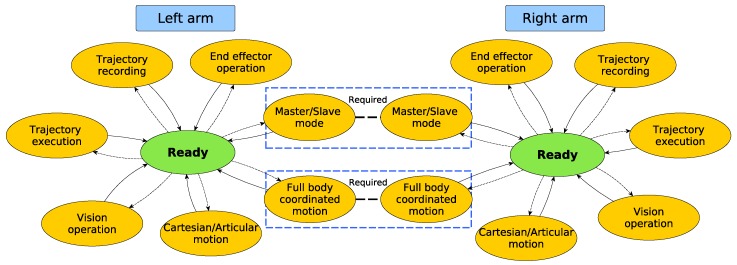
Proposed state machine-based architecture. The figure represents an overview of the architecture.

**Figure 4:**
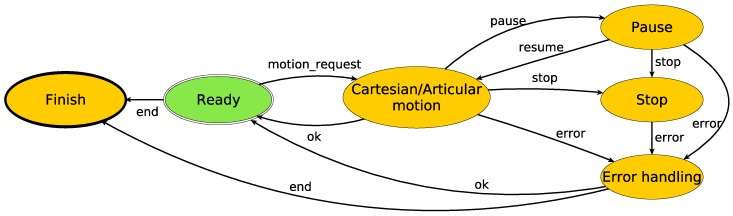
Proposed state machine-based architecture in detail. The figure shows existing states and transitions.

One of the first requirements that was identified was introspection, which is a tool able to provide the current execution state continuously, allowing us to manage possible errors and improving the recovery from them. In Figure 3 and Figure 4, the proposed architecture is outlined. The proposed architecture consists of two state machines, one per arm, with some common states. These common states are used when a synchronization between the arms is required, i.e., when both arms of the robot have to move at the same time. This combination of two state machines related by some common states combines the advantages of having individual machines for processes that do not need dual-arm cooperation, with the robustness that allows centralized states for dual-arm requiring processes. The use of the SMACH/ROS combination provides some tools that are very useful for introspection. SMACH uses ROS messages for publishing, besides other information, the current state; thus, any module of the software can be checked easily.

When the application is launched, the system starts from a ready state and keeps changing to different states that can be seen as available abilities or capacities of the robot. Note that some states have not been included in order to simplify the diagram. These states are pause/stop, error handling and finish. The proposed work in this paper allows either human or sensor-based supervision of the environment and permits canceling or adapting plans according to sensor values and perception system information. When an error occurs, e.g., in a trajectory execution, the system is able to cancel the current operation in order to handle the error and return to a safe position (if possible) or enter an alarm state that requires operator intervention.

#### 2.1.3. Description of the Developed States

Each state has been implemented as a module that generally is independent from the core. Only a few modules have been defined as fundamentals. These special modules are articular/Cartesian, full body coordinated motion and trajectory execution. All available modules for this version are shown in Figure 3. It should be emphasized that according to the requirements of the different applications, the available states can be updated by incorporating new capabilities or removing others that will not be used.

In order to understand the proposed architecture, Table 1 summarizes the different states and their utility. Besides, in Table 2, a summary of the signal and transitions is presented. Each state may contain a more or less complex structure according to its purpose. On the one hand, for example, the vision operation state only contains the calls to different vision functions. On the other hand, the articular/Cartesian motion state is highly general, i.e., this state contains all of the required code to manage motions both in Cartesian and articular spaces. For a state transitioning, different events are handle; these events can be thrown out by the safety supervision system or by any module.

### 2.2. Flexible Application Development

The proposed architecture in this paper not only refers to the state machine-based execution manager, but contains everything necessary for deploying different robotic applications. One of the key advantages of the proposed work is that different applications reuse the common structure of the framework.

#### 2.2.1. Software Structure of the Framework

In order to ease the maintainability and assure software quality, the developed framework is organized into different packages. In this way, following the ROS philosophy, each package must fulfil minimum quality criteria.

**Figure 5:**
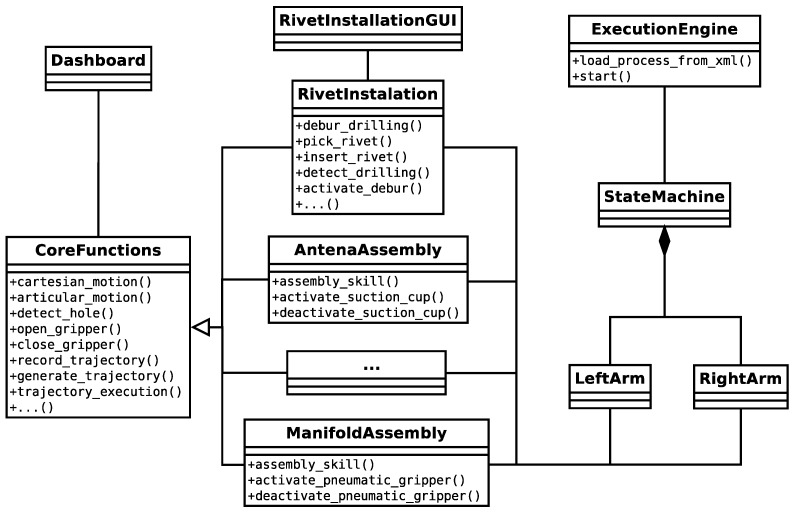
Software structure of the framework.

The simplest application is composed by at least the following three packages: execution engine, core functions and application functions. Figure 5 illustrates these packages (three columns) and the relation between them. As can be seen, the execution engine creates (instantiates) the state machines. Each state machine has an instance of an application function (RivetInstallation, AntenaAssembly, etc.). Application functions inherit from core functions all of the attributes and methods, which allows using the robot basic operations (Section 2.1.3), enhancing and particularizing them for applying into specific industrial applications. In this way, all applications are composed by core functions (basic operations) and application functions, which are a combination of the previous ones. These function libraries basically configure the requests for the state machine filling required parameters. This organization also allows having specific graphical user interfaces for each project (rivet_installation_gui) and a common one for basic robot guiding or teaching (dashboard).

#### 2.2.2. Execution Engine

The execution engine creates two threads, one per arm; these threads will contain instances of the proposed state machine. The execution engine will continue its execution managing the request of operations, i.e., the execution engine is responsible for orchestrating the application flow.

At this point, it is important to think about the change of paradigm for executing robotic applications. As has been explained here, there are three “independent” threads. As the proposed architecture is running under ROS, the state machine threads are actually ROS nodes and basically act like threads with their own parametrization and independent behavior. The execution engine communicates with these nodes via ROS messages, which contain robot commands with the necessary parametrization; in this way, each node receives commands to execute and starts triggering the state machine to the convenient state. When the operation is finished, the state machine returns to the ready state. The heart of the matter remains in how these messages are generated and managed (Section 2.2.3).

The consistency of the execution is guaranteed by the deterministic operation of the state machine. Each node will not receive the next operation until necessary synchronization requirements are met, i.e., until the execution engine can assure that state machines are in the ready state. In Section 3.1, a real use case is presented explaining how the operations are executed maintaining the coordination of both arms.

#### 2.2.3. Application to Executable XML

As mentioned above, applications are stored in XML files, with the particularity that each group of the robot (left arm, right arm and torso/head) has its own instructions. This is because each state machine needs to execute operations both synchronously and asynchronously: in some cases, a process requires both arms of the robot at the same time, e.g., a big part that needs two arms for a correct handling; in other cases, some process can require the use of both arms, but not at the same time. XML files contain, in addition to the operations, the necessary flags and synchronization tools to assure this coordination. In this paper, for the presented use case, the simplest instruction for coordination is used: a wait instruction. This allows one arm to wait until the other arm finishes its ongoing operation.

**Figure 6:**
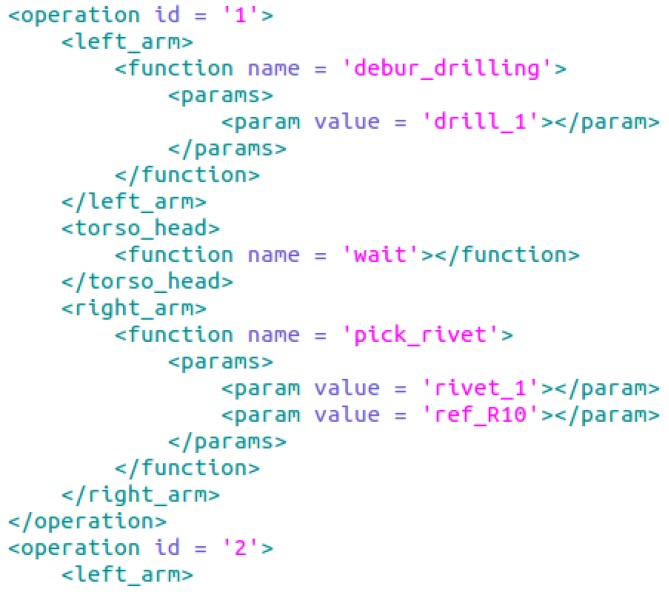
Application program fragment.

**Figure 7:**
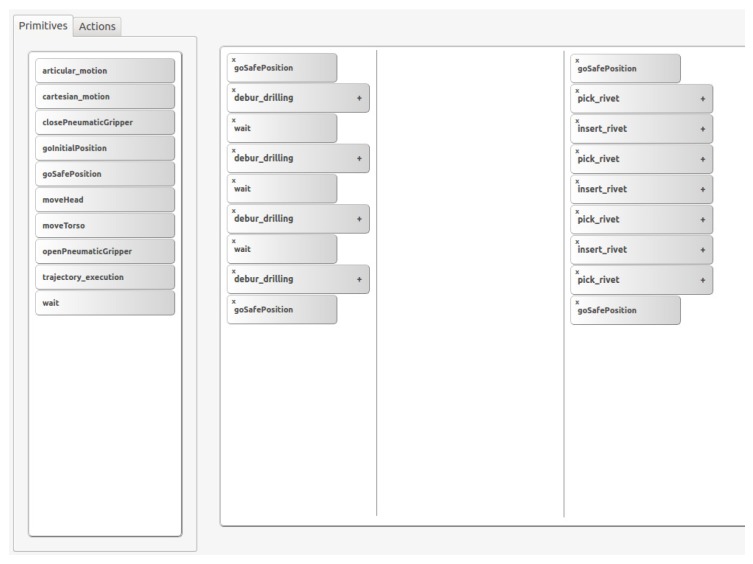
Simple GUI for new application development.

Generating a simple application (as can be seen in Figure 6) can be performed writing each XML file by hand; however, when the application and complexity grow, it is difficult to maintain the correct perspective and timeline, leading to errors. To address this, a simple graphical interface can be used. The presented GUI in Figure 7 obtains a list of available functions from core functions and application function packages (introduced in Section 2.2.1). For creating new applications, the user has to add functions and parametrize them. With the help of the graphical interface many programming errors are avoided, especially for the synchronization of both arms, allowing a global vision of the execution flow. In Figure 7, at the right frame, the application program is represented; the displayed example is for rivet installation process. When the application is ready, an XML file is created, containing the list of commands that each arm has to execute. The wait function represents the simplest synchronization mechanism, because in those time lapses, the left arm has to wait until the right arm finishes; therefore, in the generated XML file, this will be translated as the wait synchronization operation.

## 3. Results

### 3.1. Validation in a Real Use Case

As Tecnalia [31] is in direct contact with companies in different industrial sectors, these developments have been tested in several scenarios with different requirements. One of the most relevant use cases is for the aeronautics sector; Tecnalia and Airbus Operations (Puerto Real facilities, Spain) have been working together for several years developing pilot cells for a dual-arm robot (see LIAA [32] (the EU’s FP7 program) for flexible assembling operations). The first steps toward the technology transfer for industry validation of this architecture are currently in process in the Rapid Reconfiguration of Flexible Production Systems (ReCaM) (this research has received funding from the European Union’s Horizon 2020 research and innovation program under Grant Agreement No. 680759) [33] project (the EU’s Horizon 2020 program). The relation between a technological center (Tecnalia), a robotic system integrator (DGH [34]) and the end user (CESA [35]) is a key issue in ReCaM, where the aim to demonstrate a set of integrated tools for the rapid reconfiguration of flexible production systems, particularly the assembly of aeronautical actuators, is addressed.

As had been mentioned in previous work [36], one of the most relevant tasks in the aerostructure assembly is the rivet installation operation. In this paper, the progress made on the automation of the riveting installation is presented; in the current prototype a deburring operation has been added, because this prepares the surface of the drilling perimeter for the correct rivet installation. This operation is performed with an integrated deburring tool in one of the grippers of the robot. The other gripper is prepared for taking and introducing rivets into drilled holes. This demonstration takes advantage of the dual-arm capabilities. Furthermore, for robot perception, a stereo vision system has been incorporated for the precise hole detection; using incorporated stereo cameras on the arms, production tolerances (0.2 mm) can be achieved [37]. In the same way that the vision system is used, different kinds of sensors can be integrated adding the corresponding state to the state machine.

**Figure 8:**
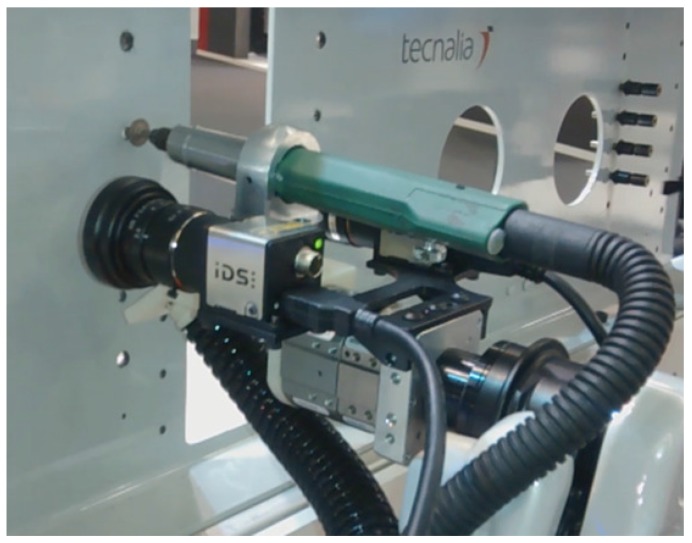
A drilled hole is deburred after detecting its position by vision.

**Figure 9:**
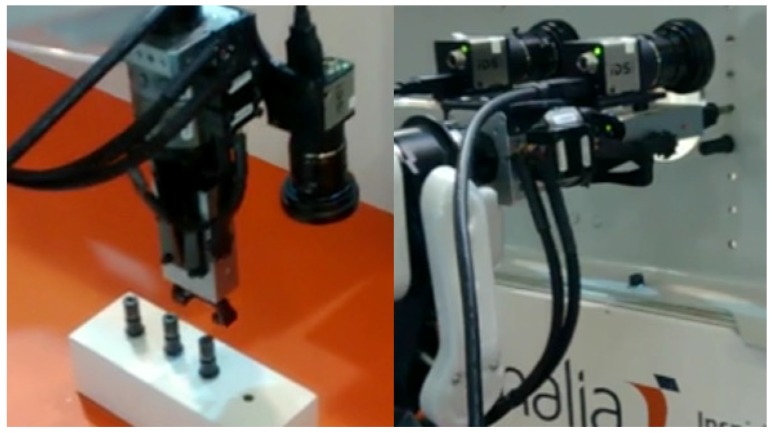
(**a**) The right arm of the robot is taking a rivet from a tray; (**b**) after taking the rivet, it is introduced in the previously-detected drilled hole.

Summarizing, the current demonstrator is composed of the following steps:

*a*. Detect and debur the drilled hole with the left arm (Figure 8).
*b*. Pick and extract the rivet from a tray with the right arm (Figure 9a).
*c*. Insert the rivet into the detected hole with the right arm (Figure 9b).

Note that Steps a and b can be performed at the same time, because the rivet extraction operation take more time than the deburring operation.

**Figure 10:**
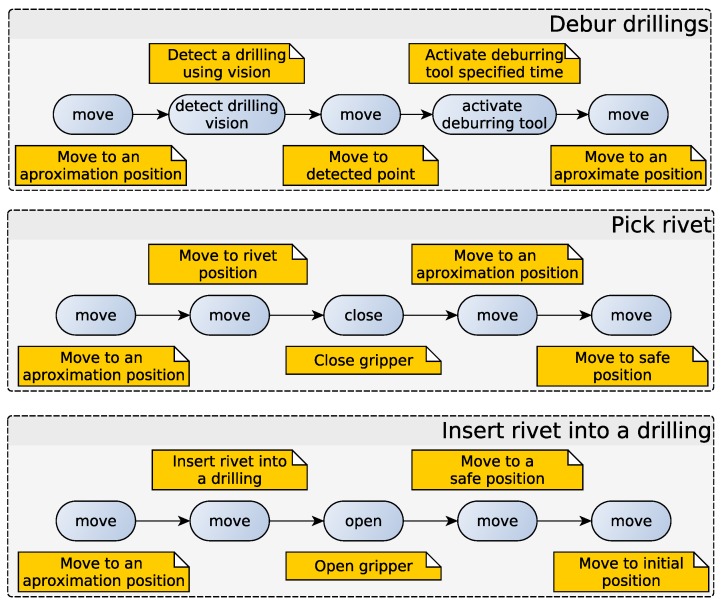
Install rivet process organized into skills. Skills are composed by primitives.

If these operations are viewed as skills, the deburring skill, pick rivet skill and rivet inserting skill are obtained. Figure 10 shows how skills are decomposed into primitives. The organization into skills eases the composition of new programs, because the parametrization is perceptibly easier. This parametrization contains the key features that vary between different skill executions. The way to determine the parameters is as follows: the system programmer starts by selecting the references or elements that change for different scenarios. For example, in the case of deburring and insertion, the references of the holes and rivets to be inserted must be parametrized. If that would not be enough, the parameters that allow one to configure the differences between scenarios would be added. Once these skills have been validated, abstracting from primitives is possible. In the case of the deburring operation, only the theoretical position must be provided, taking into account that this information can be extracted from the CAD model of the piece.

Calibration or the referencing process of the cell is beyond the scope of this work (even if it will be addressed in future work); nevertheless, it can be easily summarized in three steps: at first, the positions of the drilled holes are obtained, referenced to the origin of the CAD model. After that, using an accurate tool center point (TCP), three known points of the real piece are touched; the easiest way is usually touching one corner and their adjacent edges with the TCP. With these points, the position and orientation of the piece can be estimated. Finally, using the obtained theoretical position of the piece in the robot frame and the position of the hole in the piece frame, a frame transformation can be done to obtain the approximate position of the piece drillings. Of course, this approximate position must be corrected using artificial vision to achieve the required 0.2 mm of precision.

**Figure 11:**
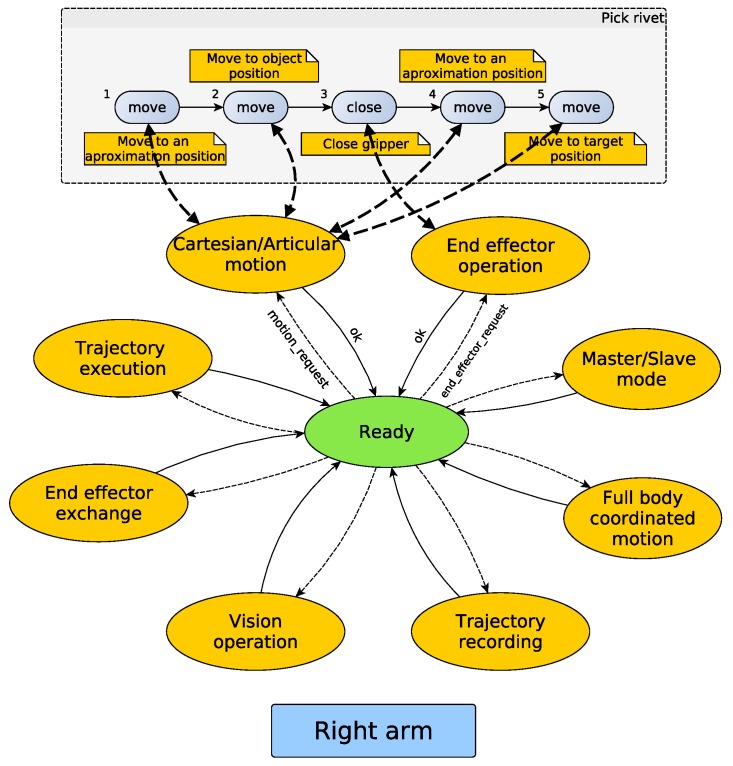
Debur drilling skill mapping into the state machine.

Returning to the proposed architecture, once the skills are decomposed, the resulting primitives are the ones that are executed by the state machine. Each state is processing the primitive callbacks and handling errors if they take place. Thus, the error handling is simpler, and it is managed specifically in each state or module. Taking one of the operations that are being analyzed, the sequence of the machine state is shown in Figure 11.

**Figure 12:**
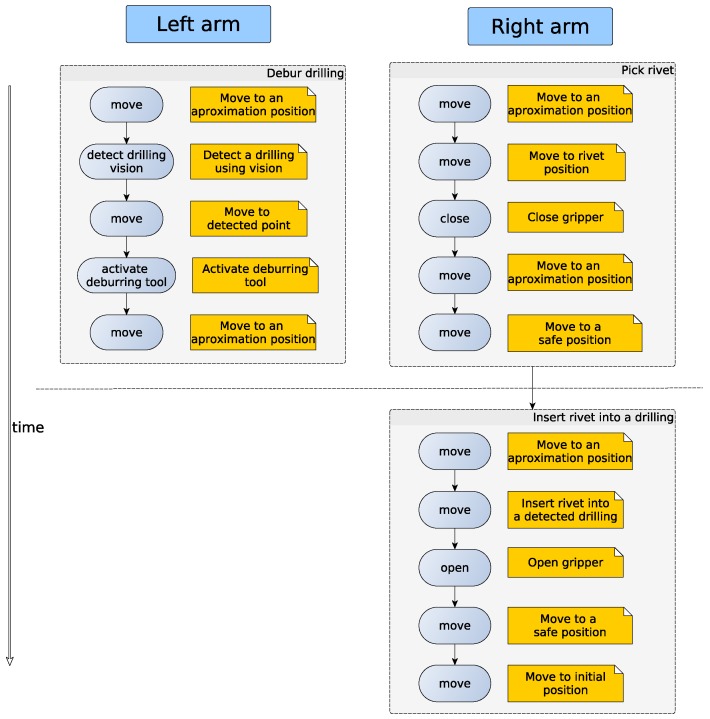
Coordination between both arms’ timeline.

The execution engine sends to the state machines the request for the next operation, based on the information that is stored in the application XML (see Figure 6). The state machine changes from one state to another, completing the requested operations. As can be seen in Figure 12, some operations of the task of installing one rivet can be performed using both arms of the robot at the same time, improving the cycle time. After these coordinated operations, an exclusive movement of the right arm is performed; at this moment, the left arm is waiting until the right arm finishes the installation of the rivet. The whole process of rivet installation is composed by the repetition of this block of skills. In order to demonstrate the adaptability of the presented framework, the CESA [35] use case is presented.

As has been mentioned above, Tecnalia is working on different projects with assembling operations in the aeronautical sector. This use case is being developed under the ReCaM project [33], one of the principal topics of which is the development of assembly capabilities for robots. In the ReCaM project, the starting point will be the product requirement description, which is first matched against the resource capabilities existing on the current system layout. If no matches are found, the system needs to be reconfigured. New resources can be searched from the resource catalogs. This matchmaking and search is allowed by the OWL-based capability model [38], which is used to describe the resource capabilities in a formal, computer- and human-interpretable manner. The capability matchmaking approach is presented in [39]. Once the system has been re-configured (or found suitable as such), the actual operations need to be programmed and executed. For this programming, the skill-based approach by Tecnalia is utilized. Basically, the required steps are the following: pick and assembly various elements (valves, springs, caps, etc.) into a manifold. All of the elements are stored in a kit, which can be referenced and located by artificial vision.

**Figure 13:**
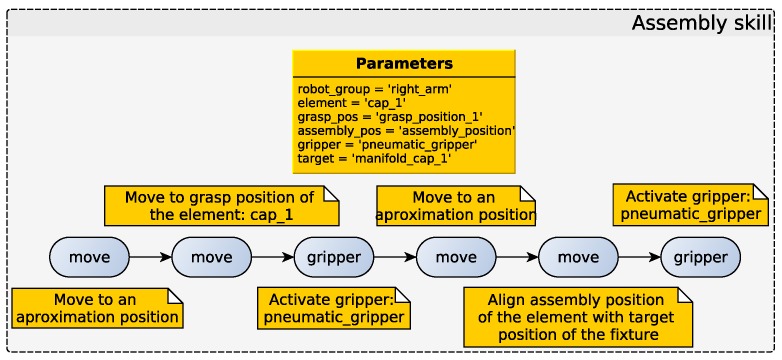
Assembly skill configuration for one cap of the manifold.

In this demonstrator, the information extracted from CAD models (an offline process that is not in the scope of this work) plays an important role. This information is modeled into different XML files: fixture information and element information. The fixture information XML file contains the position and orientation of the relevant points of the fixture; these points are marked as targets for pick and place operations. The element information XML file contains the grasp position, the necessary gripper for grasping and the assembly point in the model, i.e., the point that is necessary to align with the fixture relevant point. This skill is able to perform the steps listed above to complete the assembly of different elements into the manifold, only taking into account the information provided in the XML files. Figure 13 shows a detailed example of how the assembly skill is parametrized using the provided information.

As can be seen, different applications can be modeled following the same schema; the parameters that appear in the skill configuration are codified in an XML file (as has been presented for the previous use case in Figure 6). This XML is completely compatible with the state machine and execution engine (Section 2.1.1 and Section 2.2.2, respectively). The system capacity to adapt to changes in the environment provides advantages. For instance, if there is variation in the position of parts (elements) or in the number of parts to process, the high-level program can be adjusted through minor changes (e.g., reprocess the CAD model for updating positions and adding more blocks of a particular skill). No changes in the low-level program are needed. As a consequence, an increase of system flexibility has been achieved.

### 3.2. Evaluation

In the last few years, several methods for evaluating software architectures have been defined: scenario-based (SAAM, architecture tradeoff analysis method (ATAM), ALMA , etc.) [40,41,42,43,44,45], mathematical model-based (reliability analysis, performance analysis) [46] and metrics-based software architecture evaluation methods (QuADAI ) [47]. In order to evaluate the advantages of the proposed architecture, based on the previously-mentioned methods, a simplified approach of the architecture tradeoff analysis method (ATAM) has been selected to perform a comparative analysis [40,48,49]. This method is widely used by the research community for architecture evaluation [50,51,52]. When the architecture is evaluated, depending on the requirements, different qualities must be analyzed. ATAM concentrates on evaluating suitability; therefore, the selection of the appropriate qualities has a remarkable relevance.

As has been mentioned above, ATAM is a scenario-based method; that is why different scenarios have been selected in order to compare different desirable qualities. On the one hand, the creation of a new application from the beginning scenario has been chosen. New application deployment implies working environment definition, relevant position acquisition, fixture calibration, robot process programming, simulating, testing and adjusting. On the other hand, another common scenario is proposed, adapting an existing application to new product references (the required process would be the same, but could change the number of operations or the dimensions of the elements).

The proposed architecture has been compared with different ways of addressing the automation of an industrial process [53,54]. The traditional and most commonly-used method is online programming, i.e., teach by demonstration (moving the robot with the teach pendant), replicating the process and acquiring required way points. In other cases, the use of offline programming software can be found. This approach is composed by the following steps: the generation of the 3D scene, tag creation, trajectory planning, process planning, post-processing simulation and calibration [54]. As can be seen, the proposed framework in this article is very similar to an offline programming process, though with some improvements.

In order to evaluate different approaches, a set of desirable qualities have been analyzed:

Ease of use: To deal with the first scenario, differences between online programming and other alternatives are evident. A new application deployment requires stopping the production for fixture calibrations, way point acquisition, process replication, simulations and adjustments. These tasks require a high expertise in robotics and programming. With offline alternatives, the process can be offline almost entirely; only calibration and final adjustments require stopping the production. Generally, offline programming software is very complex and also requires highly trained staff. The cost of these technicians (plus license costs) could not be affordable for SMEs. The proposed framework provides a set of ease-to-configure primitives and skills, which reduces the training costs.

Adaptability: This quality impacts the second scenario. Modifying an existing process using online programming is very time consuming; new position acquisition moving the robot implies stopping the production. For offline programming, changes can be made without stopping the production, although depending on the nature of the changes, this could imply repeating many tasks in order to adapt the application. In the case of the proposed approach, the process is similar to offline programming, though with the particularity that the developed skills are programmed keeping in mind possible changes. For example, in the case of deburring and riveting holes (Section 3.1), possible changes in the hole positions and rivet size are anticipated, so the skill takes the information of the hole position and size from a processed CAD file. Then, the skill adapts its behavior, configuring the target position and gripper aperture with respect to the obtained information. The same idea is applied in the assembly operation; the developed skills can adapt to usual changes in this kind of process: changes in assembly points’ positions, changes in parts’ size, etc.

Reliability: The presented approach provides an implicit supervision tool: the state machine allows knowing the current status of the execution. Besides, the modular error handling permits an individualized response for the different types of errors. Traditional robot programming techniques require ad hoc error handling in each critical part of the program.

Subsetability: This is the ability to support the production of a subset of the system [54]. This concept could be important in different ways. For the commercial side, the possibility of having different optional modules (states or even skills) is an advantage. In the case of requiring incremental developments, the possibility to deliver simple prototypes that are enhanced with new modules and abilities is interesting. For the end user, having only the functionalities that are required could reduce the training time and increase the ease of use. Subsetability quality does not exist for traditional online programming, and for offline programming, software usually is used only for the commercial aspect.

Performance: Both online programming and offline programming have the best performance, because these methods do not add any layer of software in the execution time, i.e., when the configuration or set-up phase concluded, only a robot specific code is executed in the controller. In the proposed framework, the XML program is parsed for executing existing skills, which are composed by primitives that execute directly in the robot controller. This, combined with the overhead from the state machine, results in greater demands on processing resources. Even so, the executed process and robot movements are the same for all alternatives, so these differences in performance do not affect the overall operation.

Table 3 summarizes the strengths and weakness of different robot programming approaches. Online programming is the simplest approach, which only has the performance as the clear advantage. The proposed approach can be seen as an enhanced offline programming method; both have in common many insights, in spite of the fact that through the skill programming and state machine-based architecture, the ease of use, adaptability and subsetability have been improved. Thanks to the developed skills, many of assembly applications that are composed by pick and place operations can be easily modeled and resolved by the presented framework. This proposal is a step forward in the generalization of this kind of problem. These improvements have a performance drawback, but taking into account the advantages, the trade-off is acceptable.

Based on the obtained conclusions in Table 3, the representation of the claimed improvements has been done. The more important qualities that have been improved are the ease of use and the adaptability. These improvements are translated directly into the reduction of the development time. Despite that the required time for the programming of different automation processes can vary widely, one of the most usual operations has been selected: pick and place. If the CESA use case has been taken as the reference (Section 3), in the following lines, an analysis of the required time for programming the assembling operations can be found.

**Figure 14:**
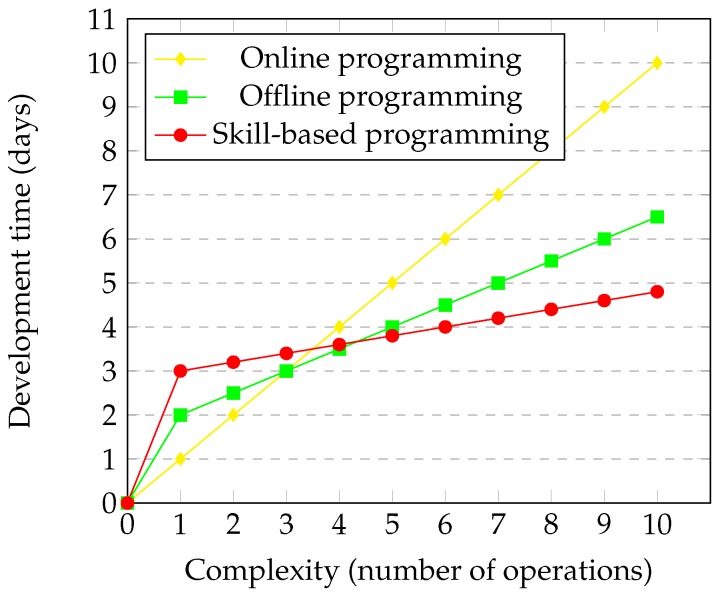
Comparison of the process development time according to its complexity.

Figure 14 shows how the online programming development time grows linearly according to the number of operations that must be programmed. Each operation requires moving the robot manually and storing waypoints. Regarding offline programming, an initial overrun can be perceived, mainly due to the required time for cell referencing, i.e., the transition between simulation and reality. After that, successive operations require less time than manual teaching. Concerning skill-based programming, higher initial overrun is necessary, due to the required cell referencing and the additional information, which complements the skills (grasp positions, assembly positions, gripper information, etc). When this information is modeled, the successive instantiation of assembly skills is faster; only drag and drop and simple parametrization are required. In conclusion, it can be perceived how when more than five operations are required, the skill-based programming offers better performance.

**Figure 15:**
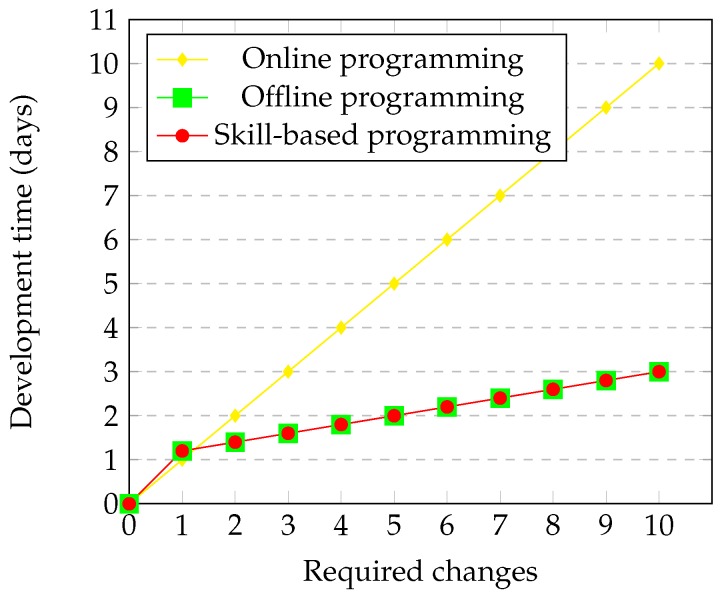
Comparison of the process development time according to the number of adjustments in element positions.

Figure 15 presents the required development time for adjusting an existing process, i.e., when something has moved or another reference of the product requires position adjustments. As before, online programming will require repeating all of the process, teaching new waypoints and assuring no collisions. Regarding offline programming and skill-based programming, in this case, they behave in a similar way: one the one hand, an initial cell referencing is necessary, and on the other hand, as the program is already created, only parameter modifications are required. Of course, the necessary changes are different in both methodologies, but the same required time has been estimated.

**Figure 16:**
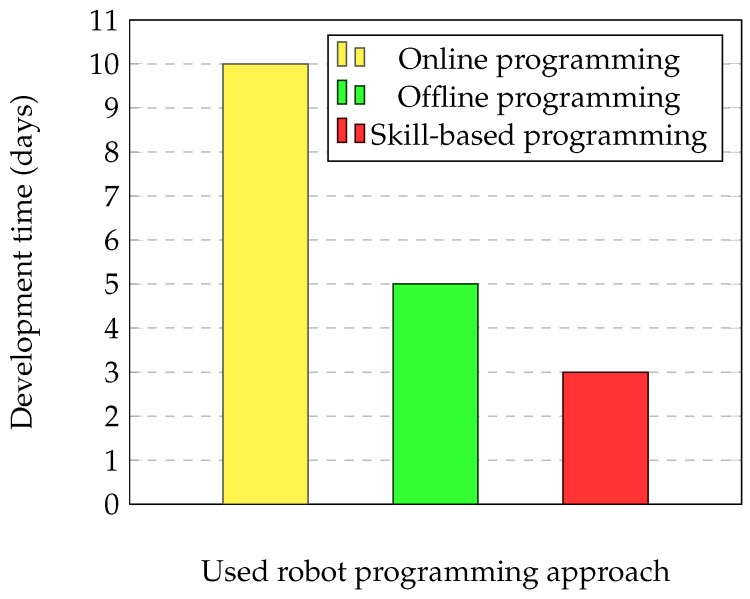
Comparison of the process development time when the robot provider is changed.

Finally, Figure 16 shows the required time if the robot of the process is changed to a different one. Taking a process composed by 10 operations, for an online programming approach, this is a completely new process. Using an offline solution, in the best case, the program sequence can be reused. However, it must be noted that a revision of all of the waypoints must be done. In the case of skill-based programming, the developed skill does not require a revision in terms of programming or parametrization because the problem to resolve is the same. For the proposed framework, this scenario is taken as another process adjustment, requiring the same time as in the previous case.

## 4. Discussion

As has been analyzed in the previous section, the presented approach in this article offers greater flexibility and reusability (adaptability) than traditional frameworks. On the one hand, the flexibility of this approach is demonstrated by the fact that the same skills can be used to perform different processes although they suffer from certain variations, e.g., variations in the rivet models, variations in the drilled holes’ number or positions, etc. This assertion is supported by the work that the authors have made in different applications [55,56,57,58]: another deburring process was performed using very similar skills; the antenna assembling skill was presented; workspace monitoring and vision operations for hole detection and 3D CAD matching were integrated as skills; and finally, the interaction between the skills and the state machine was presented. On the other hand, new applications can be generated graphically (Section 2.2.3), reducing the required expertise and increasing the ease of use. When the user adds a skill to the execution flow, all required parameters must be filled. In this way, a succession of blocks, which composes the application, is generated. The developed GUI allows exporting sections or entire applications into XML files in order to increase the re-usability.

One of the foreseen advantages of the present approach is that the state machine architecture can be enhanced with different modules (states) that could be useful in completely different processes. In the proposed scenario, the states are related to the robot primitives, i.e., robot movements controlled in velocity in the Cartesian space. Nevertheless, the proposed primitives can be combined with nonlinear controllers, such as predictive control [59], neural networks or fuzzy approaches [60,61], needed in other industrial processes with high uncertainty in the model like chemical processes (i.e., petrochemical plants). The skills approach could provide additional information and actuation; basic functionality could operate the aperture or closure of valves, and a complex implementation could cover other acting elements. This is an idea explored in the TOP-REF project [62].

Regarding reliability and robustness that the state machine provides, it permits users to abstract from the specifics of dual-arm robotic programming. The proposed framework eases the coordination of both arms with the help of a simple GUI (Figure 7). Besides, a complete traceability of the program status combined with modular error handling increases the overall reliability compared with traditional online and offline software.

One of the drawbacks of the presented approach is the performance. The entire ROS ecosystem added to the state machine requires a powerful computer, but taking into account the cost of a computer in relation to automation project costs, this is not a relevant issue. Another relevant topic is that the proposed architecture is hardware agnostic; the developed skills are not using robot-specific functions; however, when primitives are executed, ROS interfaces are used. ROS is compatible with a large number of robots [26], though for an industrial environment, ROS-Industrial [63] is more adequate. ROS-Industrial appears with the support of a large research community and robot manufacturers. Their goal is to provide reliable and robust ROS packages. The list of supported industrial robots [64] is growing day by day. This can be a disadvantage compared with available offline programming software, e.g., Delmia, which offers a huge database of robots.

In the industrial world, presenting a framework mostly composed of open source modules always causes a discussion. Even so, as has been mentioned in Section 1, nowadays, more flexibility and novel solutions are demanded, and open source initiatives like ROS are responding to these requirements of the industry.

## 5. Conclusions and Future Work

To improve the control and coordination of anthropomorphic multisensor robots, state machine-based architectures have been introduced. This approach allows us to increase the robustness and reliability of the whole system. The proposed architecture is designed to act as a basis for easier programming methodologies. Thanks to the presented graphical user interface, new applications can be generated without the need to be an expert in robotics. With the proper training, the operator will be able to create, adapt and maintain industrial processes.

Besides these advantages, the reusability has been noticeably increased. By employing the software architecture that has been presented, completely different applications can leverage well-tested modules and functions used in previous developments. At present, the same architecture is being used in different pilot stations with different types of robots and requirements; in these pilot stations, this technology is under intense tests for validating the usability, robustness and feasibility.

The proposed architecture has been compared with traditional approaches in order to analyze and highlight the strengths and weakness. ATAM has been selected in order to evaluate the qualities that have notable relevancy: ease of use, adaptability, reliability, subsetability and performance. The required development time for accomplishing assembly operations has been compared. The results of the evaluation reveal that the framework improves almost all of the mentioned qualities; the exception is the performance in terms of computational cost, which is inevitably increased by the additional software layers introduced. The next step to follow in the future will be performing a wider test bench for evaluating and comparing the performance of the robot operation with other alternatives, i.e., online and offline programming and other programming frameworks. In this evaluation, users with different levels of training could be requested. Additionally, some stress tests will be applied for assuring the stability of the system.

In future work, we will further investigate how to integrate different skill formalisms into the proposed architecture, especially for the ease of the automatic creation of new skills. The database of skills proposed in the LIAA project is another topic that will be reviewed in order to integrate more skills in the architecture. Additionally, this architecture will be integrated with the reconfigurable and flexible production system under development at ReCaM project. The tools provided by this framework will enable auto-programming and self-adjusting to the required task by utilizing parametric capabilities in the CESA use case.

Regarding the state machine-based architecture, if the proposed approach is used, the industrial processes that can benefit from dual-arm robots are more controlled, and this allows an easier and faster deployment of new applications. In the future, the focus will be set on the coordinated manipulation of the arms with the intention of easing this kind of task. Besides, the integration of a multi-agent system for decision making in coordination and synchronization tasks is being considered.

## Figures and Tables

**Table 1:** Summary of the main elements of the state machine.

State	Description
Ready	The state machine is ready for receiving new instructions. This state is waiting until the execution engine sends a new request.
Cartesian articular motion	Manages the robot movements both in the Cartesian space and the articular space. If the movement cannot be executed correctly, there is an error handling state to manage it.
Full body coordinated motion	Allows controlling both arms in coordination. Two arms must be in this state to start coordinated motion. Sending the values of the 15 joints of the robot is necessary.
Record trajectory	Allows recording trajectories with a trajectory planner or teaching by demonstration. These trajectories are stored in a database for future use.
Trajectory execution	Executes trajectories, provided by a trajectory planner or previously stored in a database.
End-effector operation	Manages end-effector operations; depending on the end effector, different operations can be made, e.g., gripper open/close, deburring tool activate/deactivate, screwing operation, etc.
Vision operation	Manages different computer vision operations. This includes picture acquisition, processing and reference frame transformation, among others. As the robotic system has multiple vision systems, this state is responsible for managing them depending on the operation that will be executed.
Master/slave mode	Puts robot in bi-manual coordinated manipulation mode; one arm actuates as the master and the other one as the slave. Consists of planning a trajectory for the master arm and then computing this trajectory with an offset for the slave arm.

**Table 2:** Summary of the signals and transitions of the state machine.

State	Signal	Transition to
Ready	motion_request	Cartesian/articular motion
	vision_request	Vision operation
	end_effector_request	End effector operation
	...	...
	end	Finish
Cartesian	ok	Ready
Articular	pause	Pause
motion	stop	Stop
	error	Error handling
Pause	resume	Cartesian/articular motion
	stop	Stop
	error	Error handling
Stop	error	Error handling
Error	ok	Ready
handling	end	Finish

**Table 3:** Strengths and weakness of different robot programming approaches.

Quality	Online Programming	Offline Programming	State Machine and Skill Based Programming Framework
Ease of use	−	+	++
Adaptability	−	+	++
Reliability	−	+−	+
Subsetability	−	+	++
Performance	++	++	−
